# Differential Long-Term Effects of First- and Second-Generation DES in Patients With Bifurcation Lesions Undergoing PCI

**DOI:** 10.1016/j.jacasi.2021.04.006

**Published:** 2021-06-15

**Authors:** Ki Hong Choi, Young Bin Song, Joo Myung Lee, Taek Kyu Park, Jeong Hoon Yang, Joo-Yong Hahn, Jin-Ho Choi, Seung-Hyuk Choi, Hyo-Soo Kim, Woo Jung Chun, Seung-Ho Hur, Seung Hwan Han, Seung-Woon Rha, In-Ho Chae, Jin-Ok Jeong, Jung Ho Heo, Junghan Yoon, Do-Sun Lim, Jong-Seon Park, Myeong-Ki Hong, Joon-Hyung Doh, Kwang Soo Cha, Doo-Il Kim, Sang Yeub Lee, Kiyuk Chang, Byung-Hee Hwang, So-Yeon Choi, Myung Ho Jeong, Soon-Jun Hong, Chang-Wook Nam, Bon-Kwon Koo, Hyeon-Cheol Gwon

**Affiliations:** a Division of Cardiology, Department of Internal Medicine, Heart Vascular Stroke Institute, Samsung Medical Center, Sungkyunkwan University School of Medicine, Seoul, Republic of Korea; b Department of Internal Medicine and Cardiovascular Center, Seoul National University Hospital, Seoul, Republic of Korea; c Division of Cardiology, Department of Internal Medicine, Samsung Changwon Hospital, Sungkyunkwan University School of Medicine, Changwon, Republic of Korea; d Division of Cardiology, Department of Internal Medicine, Keimyung University Dongsan Medical Center, Daegu, Republic of Korea; e Division of Cardiology, Department of Internal Medicine, Gachon University Gil Hospital, Incheon, Republic of Korea; f Division of Cardiology, Department of Internal Medicine, Korea University Guro Hospital, Seoul, Republic of Korea; g Division of Cardiology, Department of Internal Medicine, Seoul National University Bundang Hospital, Seongnam, Gyeonggi-do, Republic of Korea; h Division of Cardiology, Department of Medicine, Chungnam National University Hospital, Daejeon, Republic of Korea; i Division of Cardiology, Department of Internal Medicine, Kosin University Gospel Hospital, Kosin University College of Medicine, Busan, Republic of Korea; j Division of Cardiology, Department of Internal Medicine, Wonju Severance Christian Hospital, Yonsei University Wonju College of Medicine, Wonju, Republic of Korea; k Division of Cardiology, Department of Internal Medicine, Korea University Anam Hospital, Seoul, Republic of Korea; l Division of Cardiology, Department of Internal Medicine, Yeungnam University Medical Center, Daegu, Republic of Korea; m Division of Cardiology, Department of Internal Medicine, Severance Cardiovascular Hospital, Yonsei University College of Medicine, Seoul, Republic of Korea; n Division of Cardiology, Department of Internal Medicine, Inje University Ilsan Paik Hospital, Goyang, Republic of Korea; o Division of Cardiology, Department of Internal Medicine, Pusan National University Hospital, Busan, Republic of Korea; p Division of Cardiology, Department of Internal Medicine, Inje University Haeundae Paik Hospital, Goyang, Republic of Korea; q Division of Cardiology, Department of Internal Medicine, Chungbuk National University College of Medicine, Cheongju, Republic of Korea; r Division of Cardiology, Department of Internal Medicine, Seoul St. Mary's Hospital, The Catholic University of Korea, Seoul, Republic of Korea; s Division of Cardiology, Department of Internal Medicine, St. Paul's Hospital, The Catholic University of Korea, Seoul, Republic of Korea; t Division of Cardiology, Department of Internal Medicine, Ajou University Hospital, Suwon, Republic of Korea; u Division of Cardiology, Department of Internal Medicine, Chonnam National University Hospital, Gwangju, Korea

**Keywords:** bifurcation, drug-eluting stents, outcomes, percutaneous coronary intervention, BMS, bare-metal stent(s), DES, drug-eluting stent(s), MI, myocardial infarction, MV, main vessel, PCI, percutaneous coronary intervention, QCA, quantitative coronary angiography, SB, side branch, TLF, target lesion failure, TLR, target lesion revascularization

## Abstract

**Background:**

There is a paucity of data regarding the long-term clinical outcomes of first- versus second-generation drug-eluting stent (DES), especially when used to treat complex lesions such as bifurcation lesions.

**Objectives:**

The current study compares the efficacy and safety of first- versus second-generation DES at the 5-year follow-up in patients who underwent bifurcation percutaneous coronary intervention (PCI).

**Methods:**

A total of 5,498 patients with a bifurcation lesion who underwent PCI were pooled at a single patient level from COBIS (Coronary Bifurcation Stenting) registries II and III. Five-year target lesion failure (TLF) (the composite of cardiac death, myocardial infarction [MI], and target lesion revascularization [TLR]) and cardiac death or MI were compared between the use of first-generation DES (n = 2,436) and second-generation DES (n = 3,062) during PCI. Propensity score matching was performed to reduce selection bias.

**Results:**

After a 1:1 propensity score matching procedure was conducted, the cohort consisted of 1,702 matched pairs. Patients treated with second-generation DES had a significantly lower risk of TLF at 5 years than those treated with first-generation DES in both overall and propensity-matched populations (matched hazard ratio [HR_matched_]: 0.576; 95% confidence interval [CI]: 0.456 to 0.727; p <0.001). There were no significant differences in risk of a composite of cardiac death or MI between the 2 groups (HR_matched_: 0.782; 95% CI: 0.539 to 1.133, *P =* 0.193). However, among patients who required a 2-stent technique, use of the second-generation DES reduced cardiac death or MI (HR_matched_:0.422; 95% CI: 0.209 to 0.851, *P =* 0.016). On the other hand, among patients who required a one-stent technique, the risk of a composite of cardiac death or MI was similar between the 2 groups (HR_matched_: 1.046; 95% CI: 0.664 to 1.650, *P =* 0.845). There was a significant interaction between stent generation and treatment strategy for cardiac death or MI (interaction *P =* 0.029).

**Conclusions:**

In patients treated with PCI for a bifurcation lesion, the use of second-generation DES was associated with a significantly reduced risk of 5-year TLF than the use of first-generation DES. (Korean Coronary Bifurcation Stenting Registry II [NCT01642992]; COBIS II) (Korean Coronary Bifurcation Stenting Registry III [NCT03068494] COBIS III)

## Central Illustration

**Figure:**
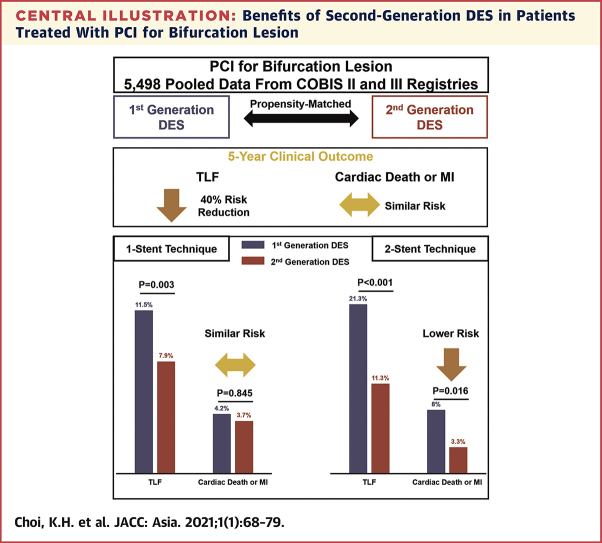

Drug-eluting stents (DES) provide gradual release of a drug to inhibit cell proliferation and markedly reduce in-stenosis restenosis after percutaneous coronary intervention (PCI) in comparison with bare-metal stents (BMS) (1,2). Nevertheless, it has been reported that there is still concern about the safety of DES; a slightly increased rate of late stent thrombosis and possibly increased rates of myocardial infarction (MI) and cardiac death after PCI (3,4). In this regard, new-generation DES with improved stent designs, thinner strut thickness, and more biocompatible polymers were developed to reduce late adverse events compared with first-generation DES. Several randomized trials were conducted to compare the efficacy and safety of first- and second-generation DES, but results among studies were conflicting, especially for long-term follow-ups (5, 6, 7, 8, 9).

PCI for bifurcation lesions is one of the most challenging and complex procedures in the field of interventional cardiology and is associated with a high risk of future adverse events, including in-stent restenosis or stent thrombosis (10,11). In particular, differences in clinical performance depending on the type of stent could be maximized when treating bifurcation lesions, because various 2-stent techniques with inevitable overlap of stents are often used during PCI. Nevertheless, there are limited data available to compare the long-term clinical outcomes of first- and second-generation DES focused on the bifurcation lesion. Therefore, this study sought to evaluate 5-year clinical outcomes of second-generation DES treatment compared with first-generation DES treatment using patient-level pooled data from COBIS (COronary BIfurcation Stenting) registries II and III, which are large-scale, bifurcation-dedicated, multicenter, real-world registries.

## Methods

### Pooled patient population

**Figure 1:**
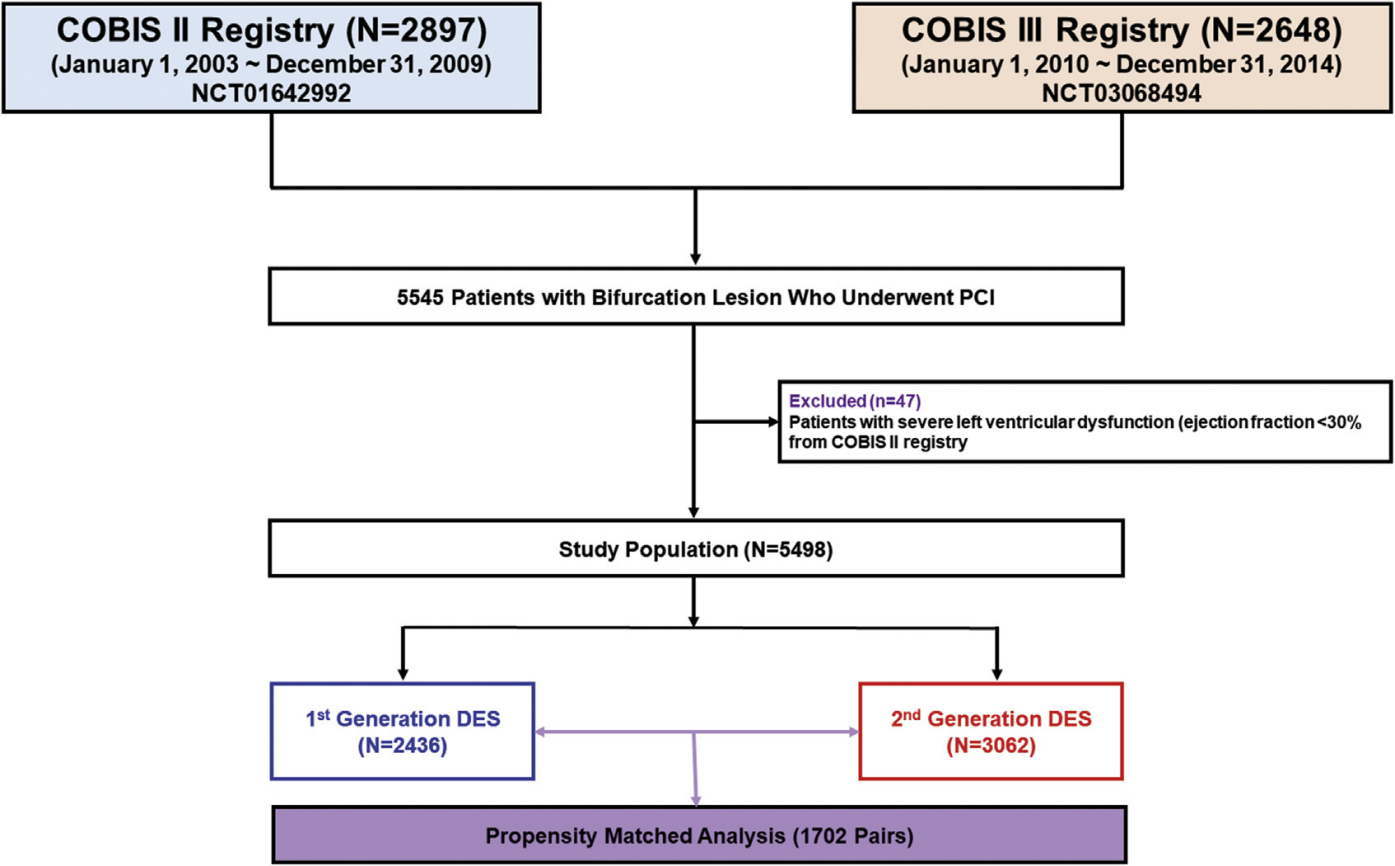
Study Flow Study flowchart of pooled data from COBIS II and III (COronary BIfurcation Stenting) registries are shown. DES = drug-eluting stent(s); PCI = percutaneous coronary intervention.

The patient-level pooled analysis included a total of 5,545 patients with bifurcation lesions who underwent PCI with DES, who were registered in the COBIS II or COBIS III registry of the Republic of Korea. Detailed individual study design and results have been documented previously (12,13). In brief, both of these registries are retrospective, multicenter, observational, dedicated bifurcation registries and had the same inclusion criteria: 1) age ≥19 years; 2) any type of coronary bifurcation lesion in the major epicardial artery treated with DES; and 3) main vessel (MV) diameter ≥2.5 mm and side branch (SB) diameter ≥2.3 mm confirmed by core laboratory quantitative coronary angiography (QCA) analysis. The COBIS II registry includes 2,897 patients treated with first- or second-generation DES from January 2003 to December 2009, whereas the COBIS III registry included 2,648 patients treated solely with second-generation DES from January 2010 to December 2014. Major exclusion criteria of both registries were cardiogenic shock or cardiopulmonary resuscitation during hospitalization and protected left main disease. In the COBIS III registry, severe left ventricular systolic dysfunction (ejection fraction <30%) was added as an exclusionary criterion. Therefore, for the present analysis, the study population was finally selected after exclusion of severe left ventricular systolic dysfunction from the COBIS II registry and stratified according to the type of stent used (Figure 1). The study protocols of both registries were approved by the institutional review board at each study center, and the requirement for written informed consent was waived due to the retrospective nature of the study. This study was conducted according to the principles of the Declaration of Helsinki.

### Percutaneous coronary intervention

Angioplasty and stenting were performed according to relevant standard guidelines at the time of each procedure (14,15). Loading doses of aspirin (300 mg) and P2Y12 inhibitors (clopidogrel: 300 to 600 mg; prasugrel: 60 mg; or ticagrelor: 180 mg) were administered before PCI, unless the patient had previously received these antiplatelet medications, regardless of stent type. Low-molecular-weight heparin or unfractionated heparin was used to achieve an activated clotting time of 250 to 300 s during the procedure. All treatment strategies, including stent type, techniques, access site, and use of intravascular imaging devices, were left to the operator’s discretion. After PCI, aspirin therapy was continued indefinitely, and the duration of the P2Y12 inhibitor prescription was at the physician’s discretion. Routine follow-up angiography was not mandatory.

### Data collection and QCA

Baseline clinical characteristics, medications, angiographic data, procedural data, and follow-up clinical outcomes were recorded using a Web-based reporting system. Additional information was obtained from medical records and telephone interviews if necessary. All coronary angiograms in both COBIS II and III registries were reviewed and analyzed quantitatively by an independent core laboratory (Heart Vascular Stroke Institute, Samsung Medical Center, Seoul, Republic of Korea) using validated software (Centricity CA 1000, GE Healthcare, Waukesha, Wisconsin). For QCA, standardized definitions for each segment of the bifurcation lesion were used as described previously: proximal MV, distal MV, and SB (16). Medina classification type 1.1.1, 1.0.1, and 0.1.1 lesions were defined as true bifurcation lesions. Bifurcation angle, minimum lumen diameter (MLD), reference vessel diameter (RD), and lesion length for each vessel were measured, and percent diameter stenosis (100 × [RD/MLD]/RD]) was calculated for each vessel.

### Study outcomes and definitions

The primary outcome was target lesion failure (TLF), a composite of cardiac death, spontaneous MI, and target lesion revascularization (TLR) at 5 years. The key secondary outcome was cardiac death or MI as a hard endpoint. Other secondary outcomes included all-cause mortality, definite stent thrombosis according to the Academic Research Consortium definition (17), and TLR. All-cause death was defined as any post-procedure death during follow-up and was considered cardiac death unless a definite noncardiac cause was established. Spontaneous MI was defined as an elevation of creatine kinase-myocardial band or troponin level greater than the upper limit of normal with concomitant ischemic symptoms or electrocardiography findings indicative of ischemia not related to the index procedure. TLR was defined as repeat PCI of the lesion within 5 mm of stent deployment. An independent clinical event-adjudicating committee, composed of independent experts in interventional cardiology who had not participated in patient enrollment, verified all clinical outcome data in both the COBIS II and III registries.

### Statistical analysis

Continuous variables are presented as mean ± SD and were compared using Welch’s *t*-test. Categorical data are summarized as numbers and relative frequencies and were compared using the chi-square test. Cumulative incidence of clinical events was studied as Kaplan-Meier estimates and compared using the log-rank test. Patients were censored at 5 years (1,825 days) or when events occurred. To identify independent predictors of TLF at 5 years, multivariate Cox proportional hazards models were used. Proportional hazards assumptions of the hazard ratios (HRs) in the Cox proportional hazards models were graphically inspected in the “log-minus log” plot and were also tested by Schoenfeld residuals. C-statistics with 95% confidence intervals (CIs) were calculated to validate the discriminant function of the model. A propensity score-matching analysis was performed to reduce selection bias and potential confounding factors. For propensity score-matching analysis, a full nonparsimonious model was developed to include all variables listed in Supplemental Table 1. Patients in the 2 groups were matched 1:1 on the logit of the propensity score with a caliper width of 0.1 of the standard deviation of the logit of the propensity score. The covariate balance after propensity score matching was assessed by calculating percentages of standardized mean differences. The absolute standardized mean difference after propensity score-matching was within ±10% across all matched covariates, demonstrating that successful balance was achieved between the comparative groups. Stratified Cox proportional hazard models were used to compare the outcomes of the matched groups. All probability values were 2-sided, and p values <0.05 were considered statistically significant. Statistical analyses were performed using R version 3.6.0 software (R Foundation, Vienna, Austria).

## Results

### Baseline characteristics and QCA analysis

Among the pooled cohort, 2,436 patients (44.3%) underwent PCI with a first-generation DES and 3,062 patients (55.7%) underwent PCI with a second-generation DES. Baseline clinical, lesion, procedural characteristics, and QCA data according to the type of stent are presented in Tables 1 and 2. Compared to patients treated with first-generation DES, those with second-generation DES were older, more likely to be male, and more commonly had general cardiovascular risk factors and complex lesion profiles, including diabetes mellitus, hyperlipidemia, current smoking, multivessel disease, and left main bifurcation (Table 1). Clinical presentation and left ventricular ejection fraction were similar between the 2 groups.

**Table 1:** Baseline Clinical and Lesion Characteristics

	First-Generation DES (n = 2,436)	Second-Generation DES (n = 3,062)	p Value	SMD
Demographics				
Age, yrs	62.1 ± 10.2	63.5 ± 11.0	<0.001	14.0
Males	1,746 (71.7)	2,315 (75.6)	0.001	8.9
Cardiovascular risk factors				
Hypertension	1,403 (57.6)	1,756 (57.3)	0.876	−0.5
Diabetes mellitus	701 (28.8)	1,027 (33.5)	<0.001	10.3
Chronic kidney disease	67 (2.8)	111 (3.6)	0.081	5.0
Hyperlipidemia	782 (32.1)	1,124 (36.7)	<0.001	9.7
Current smoking	617 (25.3)	902 (29.5)	0.001	9.3
Previous PCI	365 (15.0)	365 (11.9)	0.001	−9.0
Previous myocardial infarction	154 (6.3)	125 (4.1)	<0.001	−10.2
Previous CVA	167 (6.9)	194 (6.3)	0.473	−2.1
Initial presentation				
Clinical presentation			0.787	−1.7
Stable ischemic heart disease	931 (38.2)	1,181 (38.6)		
Unstable angina or NSTEMI	1,237 (50.8)	1,530 (50.0)		
STEMI	268 (11.0)	351 (11.5)		
LVEF %	58.8 ± 10.6	58.6 ± 9.8	0.546	−2.5
Medications at discharge				
Aspirin	2,427 (99.6)	3,016 (98.5)	<0.001	–12.4
P2Y12 inhibitors	2,412 (99.0)	3,020 (98.6)	0.237	–3.6
Clopidogrel	2,412 (99.0)	2,843 (92.8)		
Prasugrel	0 (0)	100 (3.2)		
Ticagrelor	0 (0)	79 (2.6)		
Cilostazol	630 (25.9)	422 (13.8)	<0.001	–30.9
Lesion characteristics				
Multivessel disease	1,214 (49.8)	1,850 (60.4)	<0.001	21.4
Bifurcation location			<0.001	17.3
Left main	676 (27.8)	1,095 (35.8)		
LAD/diagonal	1,347 (55.3)	1,392 (45.5)		
LCX/OM	292 (12.0)	398 (13.0)		
RCA (PL/PDA)	121 (5.0)	177 (5.8)		
Medina classification			<0.001	8.7
1.1.1	795 (32.6)	960 (31.4)		
1.0.1	186 (7.6)	192 (6.3)		
0.1.1	298 (12.2)	296 (9.7)		
1.0.0	294 (12.1)	346 (11.3)		
1.1.0	354 (14.5)	491 (16.0)		
0.1.0	416 (17.1)	661 (21.6)		
0.0.1	93 (3.8)	116 (3.8)		
True bifurcation	1,279 (52.5)	1,448 (47.3)	<0.001	−10.4

Values are mean ± SD or n (%).

CVA = cerebrovascular accident; DES = drug-eluting stent(s); LAD = left anterior descending artery; LCX = left circumflex artery; LVEF = left ventricular ejection fraction; NSTEMI = non-ST-segment elevation myocardial infarction; OM = obtuse marginal artery; PCI = percutaneous coronary intervention; PDA = posterior descending artery; PL = posterolateral artery; RCA = right coronary artery; SMD = standardized mean difference; STEMI = ST-segment elevation myocardial infarction.

**Table 2:** Baseline Procedural Characteristics and Quantitative Coronary Angiography Data

	First-Generation DES (n = 2,436)	Second-Generation DES (n = 3,062)	p Value	SMD
Treatment strategy			<0.001	−31.7
1 stent without side branch ballooning	1,010 (41.5)	1,932 (63.1)		
1 stent with side branch ballooning	768 (31.5)	580 (18.9)		
2-stent technique	658 (27.0)	550 (18.0)		
Crush	316 (13.0)	289 (9.4)		
T-stenting or TAP	239 (9.8)	157 (5.1)		
Culottes	14 (0.6)	38 (1.2)		
Kissing or V stenting	84 (3.4)	51 (1.7)		
Others	5 (0.2)	15 (0.5)		
Number of stents used	1.9 ± 1.0	1.8 ± 1.0	0.044	−5.5
Stent type			<0.001	
Everolimus-eluting stents	0 (0)	1,618 (52.8)		
Zotarolimus-eluting stents	232 (9.5)	808 (26.4)		
Biolimus-eluting stents	0 (0)	514 (16.8)		
Paclitaxel-eluting stents	806 (33.1)	0 (0)		
Sirolimus-eluting stents	1,394 (57.2)	27 (0.9)		
Mixed or other stents	4 (0.2)	95 (3.1)		
Transradial intervention	554 (22.7)	1,632 (53.3)	<0.001	66.6
Use of intravascular ultrasound	918 (37.7)	1,256 (41.0)	0.013	6.8
Final kissing ballooning	1,155 (47.4)	964 (31.5)	<0.001	−33.1
POT	514 (21.1)	813 (26.6)	<0.001	12.8
Re-POT	60 (2.5)	134 (4.4)	<0.001	10.6
NC balloon use	638 (26.2)	637 (20.8)	<0.001	−12.7
Maximal stent diameter, mm				
MV	3.2 ± 0.4	3.2 ± 0.4	0.830	−0.6
SB	2.9 ± 0.4	2.9 ± 0.4	0.092	−10.0
Stent length, mm				
MV	29.0 ± 12.2	28.6 ± 13.5	0.303	−2.8
SB	23.0 ± 10.0	21.3 ± 8.8	0.002	−18.0
Quantitative coronary angiography				
Bifurcation angle	64.3 ± 25.1	70.5 ± 22.4	<0.001	26.2
Before procedure				
MV RD, mm	3.1 ± 0.5	3.2 ± 0.5	<0.001	33.5
SB RD, mm	2.5 ± 0.4	2.6 ± 0.4	<0.001	13.9
MV MLD, mm	1.0 ± 0.5	0.9 ± 0.5	<0.001	−20.7
SB MLD, mm	1.4 ± 0.7	1.5 ± 0.8	<0.001	11.7
% of MV diameter stenosis	67.9 ± 15.6	72.7 ± 15.3	<0.001	30.7
SB % of diameter stenosis, %	46.0 ± 23.5	44.1 ± 26.8	0.005	−7.5
MV lesion length, mm	19.0 ± 12.6	18.2 ± 10.6	0.012	−7.0
SB lesion length, mm	5.5 ± 7.5	5.2 ± 6.9	0.278	−3.0
After procedure				
MV RD, mm	3.1 ± 0.5	3.3 ± 0.5	<0.001	37.8
SB RD, mm	2.5 ± 0.4	2.6 ± 0.4	<0.001	15.8
MV MLD, mm	2.7 ± 0.5	2.8 ± 0.5	<0.001	25.7
SB MLD, mm	1.8 ± 0.7	1.7 ± 0.8	0.028	−5.9
MV % of residual diameter stenosis	14.7 ± 11.6	15.4 ± 10.2	0.028	6.0
SB % of % of residual percent diameter stenosis	32.0 ± 24.3	35.6 ± 26.1	<0.001	14.1

Values are n (%) or mean ± SD.

DES = drug-eluting stent(s); IVUS = intravascular ultrasonography; MLD = minimum lumen diameter; MV = main vessel; NC = non-compliant; POT = proximal optimization technique; Re-POT = re-proximal optimization technique; RD = reference diameter; SB = side branch; SMD = standardized mean difference; TAP = T and protrusion.

With regard to the procedural characteristics for PCI of a bifurcation lesion, a one-stent crossover strategy, transradial intervention, and intravascular ultrasonography guidance were more commonly used in patients treated in the second-generation DES era than those treated in the first-generation DES era (Table 2). Specific types of stents used are listed in Supplemental Table 2. Proximal optimization technique or re-proximal optimization technique was more frequently performed, but final kissing ballooning was less frequently performed in the second-generation DES era than in the first-generation DES era. However, among patients treated with a 2-stent technique, there were no significant differences in the rates of between the first- and second-generation DES groups (first-generation DES vs. second-generation DES, 562 of 658 [85.4%] versus 484 of 550 [88.0%]; p *=* 0.218). For QCA analysis, patients treated with second-generation DES showed a higher residual percentage of diameter stenosis of the SB than those treated with first-generation DES.

After propensity score matching for the overall population, a total of 1,702 matched pairs were obtained, and significant differences of baseline clinical, lesion, procedural characteristics, as well as QCA data were balanced between the first- and second-generation DES groups (Supplemental Table 1).

### Clinical outcomes

**Figure 2:**
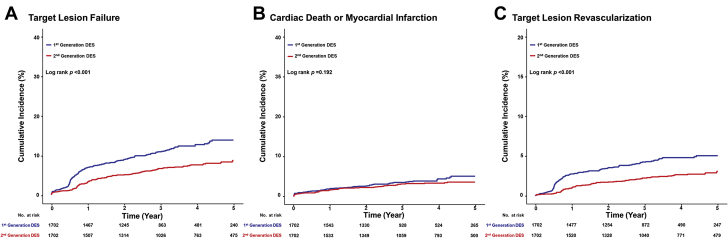
Comparison of 5-Year Clinical Outcomes According to Stent Generation in a Propensity-Matched Population Kaplan-Meier curves were used to compare the risks of target lesion failure **(A)**, cardiac death, or myocardial infarction **(B)**, and target lesion revascularization **(C)** between first- and second-generation drug-eluting stent (DES) patient groups who underwent percutaneous coronary intervention (PCI) for bifurcation lesions.

Median follow-up duration of this pooled cohort was 1,330 days (interquartile range: 844 to 1,823 days). Compared with patients with bifurcation lesions treated with first-generation DES, those treated with second-generation DES showed a significantly lower risk of TLF at 5 years (first- vs. second-generation DES: 13.1% vs. 8.5%, respectively; HR: 0.593; 95% CI: 0.494 to 0.712; p <0.001), which was mainly driven by the lower rate of TLR in the second-generation DES group (10.0% vs. 5.2%, respectively; HR: 0.457; 95% CI: 0.365 to 0.573; p <0.001) (Table 3). There were no significant differences in 5-year risk of cardiac death or MI between the first- and second-generation DES groups (4.3% vs. 4.2%, respectively; adjusted HR: 0.991; 95% CI: 0.740 to 1.327; p *=* 0.953). These results were consistent, even after multivariate Cox regression and propensity score matching analyses to adjust for baseline differences between the 2 groups (Table 3, Figure 2). Table 4 shows the multivariate Cox proportional hazard models of the total population for TLF and cardiac death or MI at 5 years. The use of second-generation DES was a powerful protective factor for TLF but not for cardiac death or MI. Among patients treated with first-generation DES, the use of sirolimus-eluting stents was associated with significantly lower risk of TLF at 5 years than with the use of paclitaxel-eluting stents (paclitaxel vs. sirolimus: 15.6% vs. 11.4%, respectively; HR: 0.619; 95% CI: 0.478 to 0.802; p <0.001) (Supplemental Figure 1).

**Table 3:** Comparison of 5-Year Risks of Clinical Outcomes According to Stent Generation

	First-Generation DES (n = 2,436)	Second-Generation DES (n = 3,062)	Univariate Analysis			Multivariate Analysis†			Propensity-Matched Analysis (1,702 Pairs)		
			HR	95% CI	p Value	HR	95% CI	p Value	HR	95% CI	p Value
TLF∗	260 (13.1)	207 (8.5)	0.593	0.494–0.712	<0.001	0.606	0.496–0.741	<0.001	0.576	0.456–0.727	<0.001
Cardiac death or MI	80 (4.3)	104 (4.2)	0.991	0.740–1.327	0.953	0.887	0.642–1.224	0.465	0.782	0.539–1.133	0.193
All–cause death	92 (5.2)	127 (5.2)	1.033	0.790–1.352	0.811	0.899	0.669–1.209	0.483	0.797	0.558–1.138	0.212
Definite stent thrombosis	13 (0.7)	19 (0.7)	1.131	0.559–2.292	0.732	1.274	0.590–2.748	0.537	1.334	0.536–3.317	0.536
TLR	200 (10.0)	123 (5.2)	0.457	0.365–0.573	<0.001	0.503	0.394–0.642	<0.001	0.527	0.400–0.695	<0.001

Values are n (%) unless otherwise indicated. Cumulative incidence rates of events are Kaplan-Meier estimates.

CI = confidence interval; DES = drug-eluting stent(s); HR = hazard ratio; MI = myocardial infarction; TLR = target lesion revascularization; TLF = target lesion failure.

∗ TLF was defined as the composite of cardiac death, MI, and TLR.

† Adjusted variables included age, sex, hypertension, diabetes mellitus, chronic kidney disease, hyperlipidemia, previous MI, previous percutaneous coronary intervention, acute coronary syndrome, left ventricular ejection fraction, multivessel disease, true bifurcation, transradial intervention, left main bifurcation, use of intravascular ultrasonography, use of a two-stent technique, final kissing balloon, proximal optimization technique, pre-main vessel percent diameter stenosis, and post-side branch percent diameter stenosis.

**Table 4:** Independent Predictors of Clinical Outcomes at 5 Years

	Adjusted HR (95% CI)∗	p Value
Target lesion failure		
Second generation DES	0.606 (0.496–0.741)	<0.001
Left ventricular ejection fraction (per 1-yr increase)	0.987 (0.977–0.996)	0.006
Final kissing ballooning	0.748 (0.589–0.949)	0.017
Transradial intervention	0.758 (0.612–0.940)	0.012
Left main bifurcation	1.526 (1.228–1.896)	<0.001
2-stent technique	1.981 (1.489–2.636)	<0.001
Age (per 1-yr increase)	1.010 (1.001–1.019)	0.035
Males	1.292 (1.036–1.613)	0.023
Diabetes mellitus	1.325 (1.091–1.610)	0.005
Chronic kidney disease	2.535 (1.802–3.566)	<0.001
Cardiac death or myocardial infarction		
Second–generation DES	0.887 (0.642–1.224)	0.465
Left ventricular ejection fraction (per 1-yr increase)	0.982 (0.968–0.996)	0.012
Left main bifurcation	1.931 (1.353–1.755)	<0.001
2-stent technique	1.763 (1.084–2.868)	0.022
Age (per 1-yr increase)	1.032 (1.017–1.047)	<0.001
Chronic kidney disease	3.604 (2.259–5.751)	<0.001
Previous myocardial infarction	1.790 (1.003–3.195)	0.049
Acute coronary syndrome	1.762 (1.255–2.475)	0.001

Abbreviations as in Table 3.

∗ C-index of the Cox regression model was 0.675 (95% CI: 0.650 to 0.701) for TLF and 0.724 (95% CI: 0.687 to 0.761) for cardiac death or MI.

**Figure 3:**
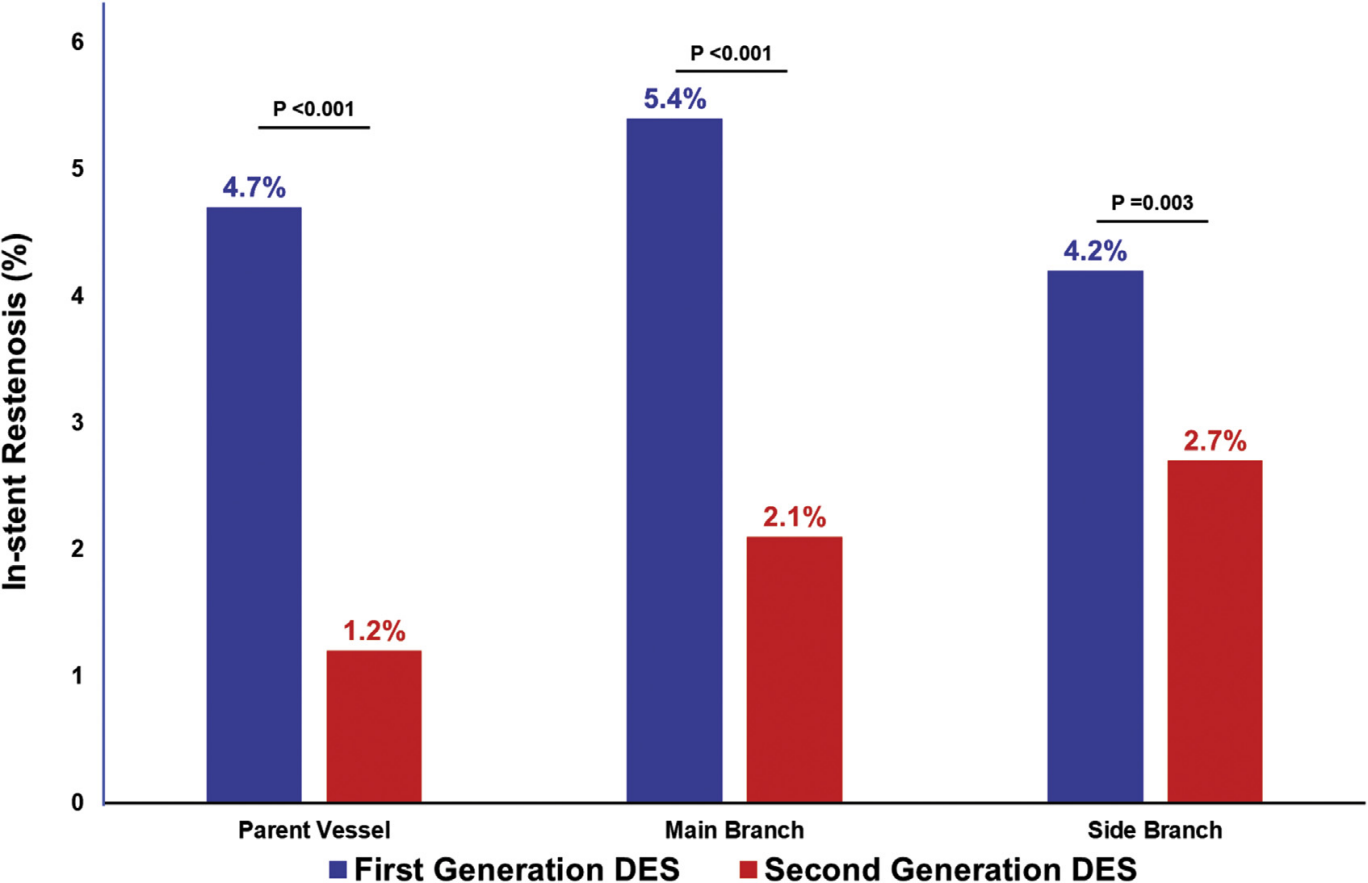
Locations of In-Stent Restenosis in the First- and Second-Generation DES Groups Bar graphs show the proportion of in-stent restenosis during follow-up in the parent vessel, main branch, and side branch in first-generation drug-eluting stent (DES) **(blue bars)** and second-generation DES **(red bars)** groups.

Figure 3 shows the specific location of in-stent restenosis lesions during the follow-up period. In patients who underwent PCI for a bifurcation lesion using first-generation DES, main branch in-stent restenosis lesions were the lesions most frequently observed. In patients treated with second-generation DES, in-stent restenosis in the side branch was the lesion most frequently observed. In-stent restenosis in all locations was significantly lower in the second-generation DES era than in the first-generation DES era.

### Subgroup analysis

**Figure 4:**
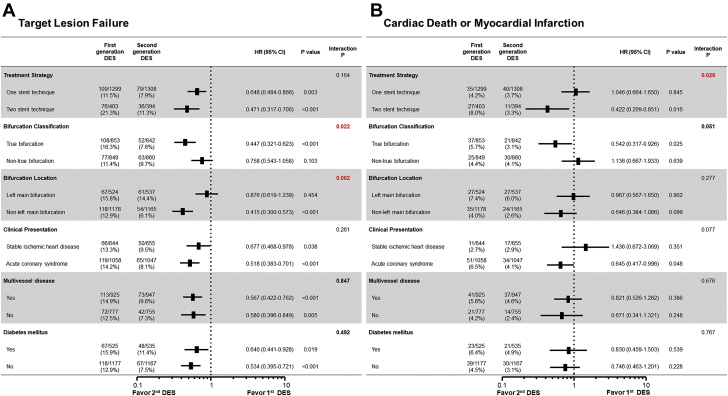
Subgroup Analysis in the Propensity-Matched Population Comparative hazard ratios of target lesion failure **(A)** and cardiovascular death or myocardial infarction **(B)** at 5 years for various subgroups in the propensity-matched population who underwent percutaneous coronary intervention (PCI) for bifurcation lesions. **Red text** denotes statistically significant differences. CI = confidence interval; HR = hazard ratio.

In exploratory subgroup analysis of the propensity-matched population, TLF and cardiac death or MI were compared between first- and second-generation DES groups according to various clinical, lesion, and procedural characteristics (Figure 4). For TLF, the beneficial effects of second-generation DES, compared with first-generation DES, were more prominent in patients with a true bifurcation lesion (interaction p *=* 0.022) (Figure 4A). Among patients with a non-left-main bifurcation lesions, the use of second-generation DES significantly reduced the risk of TLF compared to first-generation DES (12.9% vs. 6.1%, respectively; HR_matched_: 0.415; 95% CI: 0.300 to 0.573; p <0.001) but not patients with a left-main bifurcation (15.8% vs. 14.4%, respectively; HR_matched_: 0.876; 95% CI: 0.619 to 1.239; p *=* 0.454). There was a significant interaction between the type of DES and bifurcation location (left-main versus non-left-main bifurcation) for TLF (interaction p *=* 0.002) (Figure 4A). The use of second-generation DES was associated with a significantly lower risk of cardiac death or MI at 5 years in patients who underwent PCI using a 2-stent technique (8.0% vs. 3.3%, respectively; HR_matched_: 0.422; 95% CI: 0.209 to 0.851; p *=* 0.016) but not those who underwent PCI using a one-stent technique (4.2% vs. 3.7%, respectively; HR_matched_: 1.046; 95% CI: 0.664 to 1.650; p *=* 0.845), with a significant interaction (p = 0.029) (Figure 4B).

## Discussion

In the current study, we performed a patient-level pooled analysis of 2 large dedicated bifurcation registries to compare 5-year clinical outcomes between first- and second-generation DES. Our main findings were as follows: although the proportion of higher-risk patients with more complex lesions who were treated with PCI for a bifurcation lesion tended to increase in the era of second-generation DES, the stenting strategy became simpler in the second-generation DES era than in the first-generation DES era. Nevertheless, patients who underwent bifurcation PCI with second-generation DES had significantly lower risks of TLF and TLR at 5 years than those who underwent stenting with first-generation DES. However, the incidence of cardiac death or MI did not differ according to stent type. The effects of second-generation DES on the reduction of TLF were more prominent in patients with a true bifurcation lesion or non-left-main bifurcation lesion. In particular, the use of second-generation DES significantly reduced the risk of cardiac death or MI compared with the use of first-generation DES in patients who underwent bifurcation PCI using a 2-stent technique (Central Illustration).

**Central Illustration:**
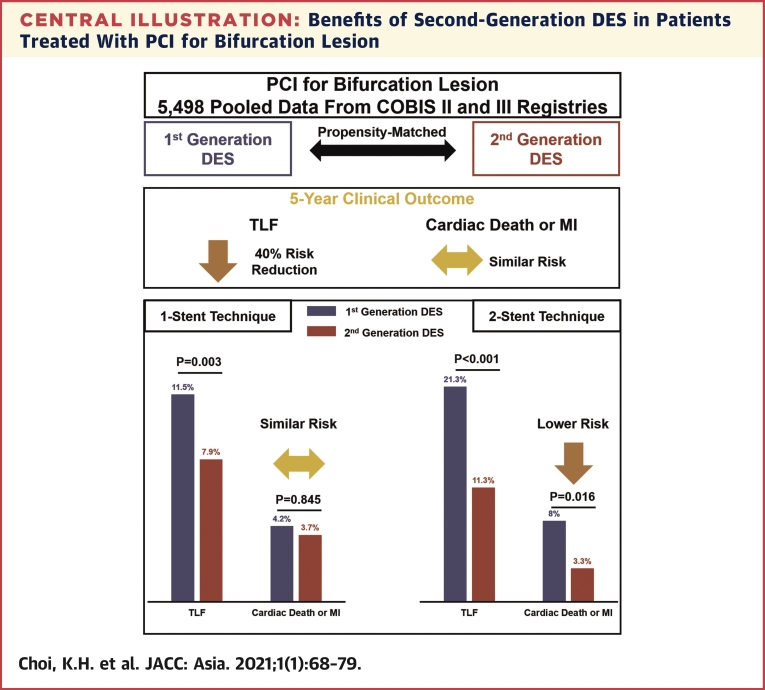
Benefits of Second-Generation DES in Patients Treated With PCI for Bifurcation Lesion This study compared the long-term efficacy and safety of the first- versus the second-generation drug-eluting stent (DES) in patients with a bifurcation lesion who underwent percutaneous coronary intervention (PCI), using patient-pooled data from the COBIS II and III (COronary BIfurcation Stenting ) registries. The use of second-generation DES was associated with a 40% risk reduction of target lesion failure (TLF) compared with the use of first-generation DES. However, there were no significant differences in hard endpoints such as cardiac death or myocardial infarction (MI), between the 2 groups. After stratifying into the stent technique, the risk of cardiac death or MI was only significantly lower in patients treated with the 2-stent technique with second-generation DES than in those with first-generation DES. There was a significant interaction between the type of DES and stent strategies for cardiac death or MI (interaction *P* = 0.029).

### Changes in treatment strategy for bifurcation lesions from the first- to second-generation DES eras

Since the introduction of DES, PCI procedures have been evolving rapidly, and better outcomes have been achieved over the last 2 decades than when DES stents were first introduced. Previous real-world registries demonstrated that the performance of PCI for patients with complex lesion characteristics or higher clinical risk profiles increased significantly over time (18,19). Similarly, our dedicated pooled bifurcation registry showed older and sicker patients with complex lesion profiles received PCI more frequently in the second-generation DES era than in the first-generation DES era. This may be because the improved stent profile of second-generation DES has allowed interventional cardiologists to perform more complex PCI procedures. Nevertheless, the shift from first- to second-generation DES simplified the treatment strategy for bifurcation PCI. In fact, the current study showed that the 2-stent technique was performed in only 17% of the study population in the second-generation era. However, 27% of the study population received a 2-stent technique in the first-generation DES era. As a result, it seems that the recurrence of second-generation DES was relatively high in the side branch, where the option to leave it alone or ballooning only was performed. This tendency may also have been influenced by the results of previous randomized controlled trials that compared outcomes between provisional one-stent strategy and elective 2-stent strategy in bifurcation PCI procedures (20, 21, 22, 23, 24, 25). We therefore performed strict adjustment of baseline clinical, lesion, and procedural characteristics through propensity score matching analysis to accurately compare efficacy and safety between first- and second-generation DES for the treatment of bifurcation lesions.

### Long-term clinical outcomes of first- versus second-generation DES in bifurcation lesions

Although second-generation DES are known to be more biocompatible and less thrombogenic than first-generation DES due to their thinner strut and improved polymers, the long-term safety and efficacy of first- versus second-generation DES remains controversial. Recently, the RESET (Randomized Evaluation of sirolimus-Eluting Versus Everolimus-Eluting Stent Trial) demonstrated that the risk of TLR was not significantly different between second-generation everolimus-eluting stents (EES) and first-generation sirolimus-eluting stents (SES) over 7 years of follow-up (8). A recent pooled analysis of 19 randomized trials also showed that late stent-related events (1 to 5 years) from BMS were similar to those in contemporary DES (26). In contrast, the ISAR-TEST 4 (Intracoronary Stenting and Angiographic Results: Test Efficacy of 3 Limus-Eluting Stents), which compared second-generation biodegradable polymer-based SES with second-generation permanent polymer-based EES with first-generation SES reported that first-generation SES were associated with significantly higher rates of adverse events including definite stent thrombosis than with second-generation biodegradable polymer-based SES or second-generation permanent polymer-based EES during 10 years of follow-up (5). One of the possible explanations for this discrepancy may be the different clinical and lesion profiles of the enrolled populations in the different studies. In fact, the ISAR-TEST 4 trial population appeared to have more complex clinical and lesion profiles than the RESET trial population, although a direct comparison of baseline differences between the 2 studies is difficult. By focusing on bifurcation lesions using the current pooled registry, the difference between first- and second-generation DES might have been maximized.

Burzotta et al. (27) previously conducted a randomized controlled trial to compare outcomes between first-generation SES and second-generation EES for the treatment of bifurcation lesions using a provisional approach and demonstrated that EES had similar procedural performance and clinical outcomes as SES but better 3-dimensional QCA results in the SB. However, only patients treated with the provisional approach were included in the study, which also had a limited sample size (n = 150) and a relatively short-term follow-up period (18 months). Moreover, previous analysis of the COBIS II registry showed comparable clinical outcomes between first-generation SES and second-generation EES (28). In contrast to these previous studies, we used the largest dedicated bifurcation pooled registry (n = 5,498) studied to date and demonstrated a significant reduction in TLF and TLR at 5 years in the second-generation DES era compared to the first-generation DES era, even after performing propensity score matching analysis to adjust for baseline differences. This result implies that clinical outcomes can be affected by type of stent when performing PCI for lesions with complex profiles, such as bifurcation lesions.

### Differential effects of stent design according to lesion and procedural characteristics

Unlike other coronary lesions, lesion location (left-main bifurcation vs. non-left-main bifurcation), lesion characteristics according to the Medina classification, and various stent strategies should be additionally considered by the interventional cardiologist during PCI performed for a bifurcation lesion. Differences in clinical outcomes according to these lesion or procedural characteristics have already been well documented in previous studies (13,29,30). Therefore, we performed subgroup analyses to evaluate if second-generation DES versus first-generation DES had different effects according to various lesion or procedural characteristics. We found that second-generation DES, compared to first-generation DES, were more effective at reducing TLF in true bifurcation lesions. Surprisingly, we also found that the reduction in risk of TLF in patients treated with second-generation DES was more pronounced for non-left-main bifurcation lesions than for left-main bifurcation lesions, in contrast to previous results from the COBIS II registry (28). Considering the results of the BASKET–PROVE (Basel Stent Kosten-Effektivitäts Trial-Prospective Validation Examination) trial, which showed comparable outcomes for first-generation SES and second-generation EES in PCI of large coronary arteries (9), the discrepancy in the benefits of second-generation DES according to lesion location among studies might have been due to differences in the size of the stent used in left-main versus non-left-main bifurcations. Furthermore, left-main bifurcations showed a higher rate of relapse after PCI due to the intrinsic characteristics of these lesions. Indeed, our study showed a high cumulative incidence for TLF (14.4%) at 5 years for left-main bifurcations, even when second-generation DES were used. Therefore, efforts to reduce stent-related adverse events after PCI, especially for left main bifurcations, are needed, even in the current second-generation DES era. We also found that cardiac death or MI in the overall population with bifurcation lesions was not significantly different between the first- and second-generation DES era. However, compared with first-generation DES, the use of second-generation DES was associated with a significantly lower risk of cardiac death or MI in patients with a bifurcation lesion who underwent PCI using a 2-stent strategy. This result suggests that an improved stent profile may be more critical for reducing the risk of hard endpoints when performing complex bifurcation PCI using a 2-stent technique where stents overlap, and morphology changes are inevitable.

### Study limitations

First, it is possible that unmeasured confounding factors influenced the study results due to our use of data from an observational registry. In particular, changes in trends regarding the treatment of bifurcation lesions over time might have be a source of potential bias in the current study, even though propensity score matching analysis was performed to adjust for baseline differences. Furthermore, treatment strategies and performance of follow-up angiography were left to the physician’s preference, and it might have affected the follow-up outcomes. Second, although the current analysis was performed using the largest bifurcation-dedicated pooled registry assembled to date, sample sizes were inadequate to analyze differences in stent thrombosis risks between the 2 groups. Finally, although minimized exclusion criteria were applied in both the COBIS II and COBIS III registries to reflect real-world practice, patients with cardiogenic shock, cardiopulmonary resuscitation, and severe left ventricular dysfunction were excluded. In this regard, differences in acute stent-related events immediately after PCI between first- and second-generation DES might not be reflected in the findings of the current study.

## Conclusions

Analysis of a large, patient-level-dedicated bifurcation pooled cohort of 5,498 patients treated with PCI identified the fact that, at the 5-year follow-up, use of second-generation DES was associated with a significantly lower risk of TLF, mainly driven by a reduction in TLR, compared with the use of first-generation DES. Overall, the risk of cardiac death or MI did not differ between the first- and second-generation DES era. However, the use of second-generation DES was associated with a significantly lower risk of cardiac death or MI in patients who required a 2-stent technique for a bifurcation lesion. It should be interpreted with caution because changes in trends of treatment strategy and more experience regarding the bifurcation PCI over time are some of the potential biases.

Perspectives **COMPETENCY IN MEDICAL KNOWLEDGE:** After the introduction of the second-generation DES, the rates of device-related failure or target lesion failure such as restenosis and stent thrombosis have been markedly decreased compared with the rates from the era of bare-metal stents or first-generation DES. However, long-term results between the first- and second-generation DES are still conflicting, and there are limited data focused on the treatment of complex lesions such as bifurcation lesions.

**COMPETENCY IN PATIENT CARE:** The use of second-generation DES was associated with significantly lower long-term risk of TLF compared with the use of first-generation DES in patients with bifurcation lesion who underwent PCI. The beneficial effects of second-generation DES on long-term clinical outcomes were more prominent in patients with a true bifurcation lesion or a non-left-main bifurcation lesion, or who were treated using a 2-stent technique.

**TRANSLATIONAL OUTLOOK:** It should be noted that PCI performed for a left main bifurcation showed a high risk of TLF, even in the era of second-generation DES. There is a need to reduce the risk of TLF after PCI for left main bifurcation lesion through the development of new devices, techniques, or medical treatment.

## Funding Support and Author Disclosures

Supported by the Korean Bifurcation Club (COBIS III) and Korean Society of Interventional Cardiology (COBIS II and III). The authors have reported that they have no relationships relevant to the contents of this paper to disclose.
