# Supplementary material for: Differential Long-Term Effects of First- and Second-Generation DES in Patients With Bifurcation Lesions Undergoing PCI
Source: JACC Asia. 2021 Jun 15;1(1):68–79. doi: 10.1016/j.jacasi.2021.04.006 (PMC9627880; doi:10.1016/j.jacasi.2021.04.006)
Supplement: Supplemental Tables 1 and 2 and Supplemental Figure 1 [file mmc1.docx]

**Supplemental Table 1. Baseline clinical, lesion, procedural characteristics, and quantitative coronary angiography data in propensity matched population**

|  | **First generation DES (n=1702)** | **Second generation DES (n=1702)** | **P value** | **SMD** |
| --- | --- | --- | --- | --- |
| Age (years) | 62.7±10.2 | 62.9±11.1 | 0.656 | 1.5 |
| Male sex | 1254 (73.7%) | 1248 (73.3%) | 0.846 | -0.8 |
| Hypertension | 983 (57.8%) | 1000 (58.8%) | 0.578 | 2.0 |
| Diabetes mellitus | 525 (30.8%) | 535 (31.4%) | 0.739 | 1.2 |
| Chronic kidney disease | 54 (3.2%) | 59 (3.5%) | 0.702 | 1.6 |
| Hyperlipidemia | 582 (34.2%) | 588 (34.5%) | 0.857 | 0.7 |
| Current smoking | 474 (27.8%) | 467 (27.4%) | 0.818 | -0.9 |
| Previous PCI | 219 (12.9%) | 229 (13.5%) | 0.648 | 1.8 |
| Previous myocardial infarction | 82 (4.8%) | 85 (5.0%) | 0.874 | 0.9 |
| Previous CVA | 115 (6.8%) | 121 (7.1%) | 0.736 | 1.5 |
| Clinical presentation |  |  | 0.876 | -0.6 |
| Stable ischemic heart disease | 644 (37.8%) | 655 (38.5%) |  |  |
| Unstable angina or NSTEMI | 854 (50.2%) | 839 (49.3%) |  |  |
| STEMI | 204 (12.0%) | 208 (12.2%) |  |  |
| LVEF (%) | 58.8 ± 9.5 | 58.8 ± 9.1 | 0.915 | 0.4 |
| Aspirin | 1694 (99.5%) | 1696 (99.6%) | 0.789 | 1.0 |
| P2Y12 inhibitors^*^ | 1691 (99.4%) | 1692 (99.4%) | >0.999 | 0.5 |
| Cilostazol | 343 (20.2%) | 317 (18.6%) | 0.278 | -4.4 |
| Multi-vessel disease | 925 (54.3%) | 947 (55.6%) | 0.469 | 2.6 |
| Left main bifurcation | 524 (30.8%) | 537 (31.6%) | 0.657 | 1.6 |
| True bifurcation | 853 (50.1%) | 842 (49.5%) | 0.732 | -1.3 |
| Two stenting technique | 403 (23.7%) | 394 (23.1%) | 0.746 | -1.4 |
| No. of used stent | 1.8±1.0 | 1.9±1.0 | 0.671 | 1.5 |
| Trans-radial intervention | 540 (31.7%) | 542 (31.8%) | 0.971 | 0.2 |
| IVUS guidance | 687 (40.4%) | 690 (40.5%) | 0.944 | 0.4 |
| Final kissing ballooning | 674 (39.6%) | 673 (39.5%) | >0.999 | -0.1 |
| POT | 406 (23.9%) | 398 (23.4%) | 0.778 | -1.1 |
| Re-POT | 54 (3.2%) | 52 (3.1%) | 0.921 | -0.6 |
| NC balloon use | 419 (24.6%) | 422 (24.8%) | 0.937 | 0.4 |
| Maximal MV stent diameter, mm | 3.2±0.4 | 3.2±0.4 | 0.928 | -0.3 |
| Maximal MV stent length, mm | 29.0±12.2 | 29.0±14.0 | 0.909 | -0.4 |
| Bifurcation angle | 66.9±26.2 | 67.6±21.7 | 0.356 | 3.4 |
| Pre MV percent diameter stenosis, % | 69.7±15.2 | 70.1±16.1 | 0.450 | 2.6 |
| Pre SB percent diameter stenosis, % | 45.5±23.7 | 45.1±26.6 | 0.620 | -1.6 |
| MV residual percent diameter stenosis, % | 15.0±11.9 | 15.0±10.6 | 0.909 | 0.4 |
| SB residual percent diameter stenosis, % | 33.6±24.6 | 33.4±26.0 | 0.797 | -0.9 |

Data are presented as mean ± standard deviation or n (%).

^*^P2Y12 inhibitors included clopidogrel, ticagrelor, and prasugrel.

Abbreviations: CVA=cerebrovascular accident; DES=drug-eluting stent; IVUS=intravascular ultrasound; LVEF=left ventricular ejection fraction; MV=main vessel; NC=non-compliant; NSTEMI=non-ST-segment elevation myocardial infarction; PCI=percutaneous coronary intervention; POT=proximal optimization technique; SB=side branch; SMD=standardized mean difference; STEMI=ST-segment elevation myocardial infarction.

**Supplemental Table 2. Specific type of used stent**

| **First generation DES (n=2436)** | **Second generation DES (n=3062)** |
| --- | --- |
| Paclitaxel eluting stent | Everolimus eluting stent |
| Taxus (n=760) | Xience prime/V/expedition (n=1145) |
| Pico elite (n=25) | Promus premier/element (n=473) |
| Coroflex please (n=21) | Zotarolimus eluting stent |
| Sirolimus eluting stent | Endeavor resolute (n=72) |
| Cypher (n=1394) | Resolute integrity (n=736) |
| Zotarolimus eluting stent | Biolimus eluting stent |
| Endeavor (n=232) | Biomatrix/Novori (n=514) |
| Others or mixed (n=4) | Sirolimus eluting stent |
|  | Orsiro (n=27) |
|  | Others or mixed (n=95) |

Abbreviations: DES=drug-eluting stent.

**
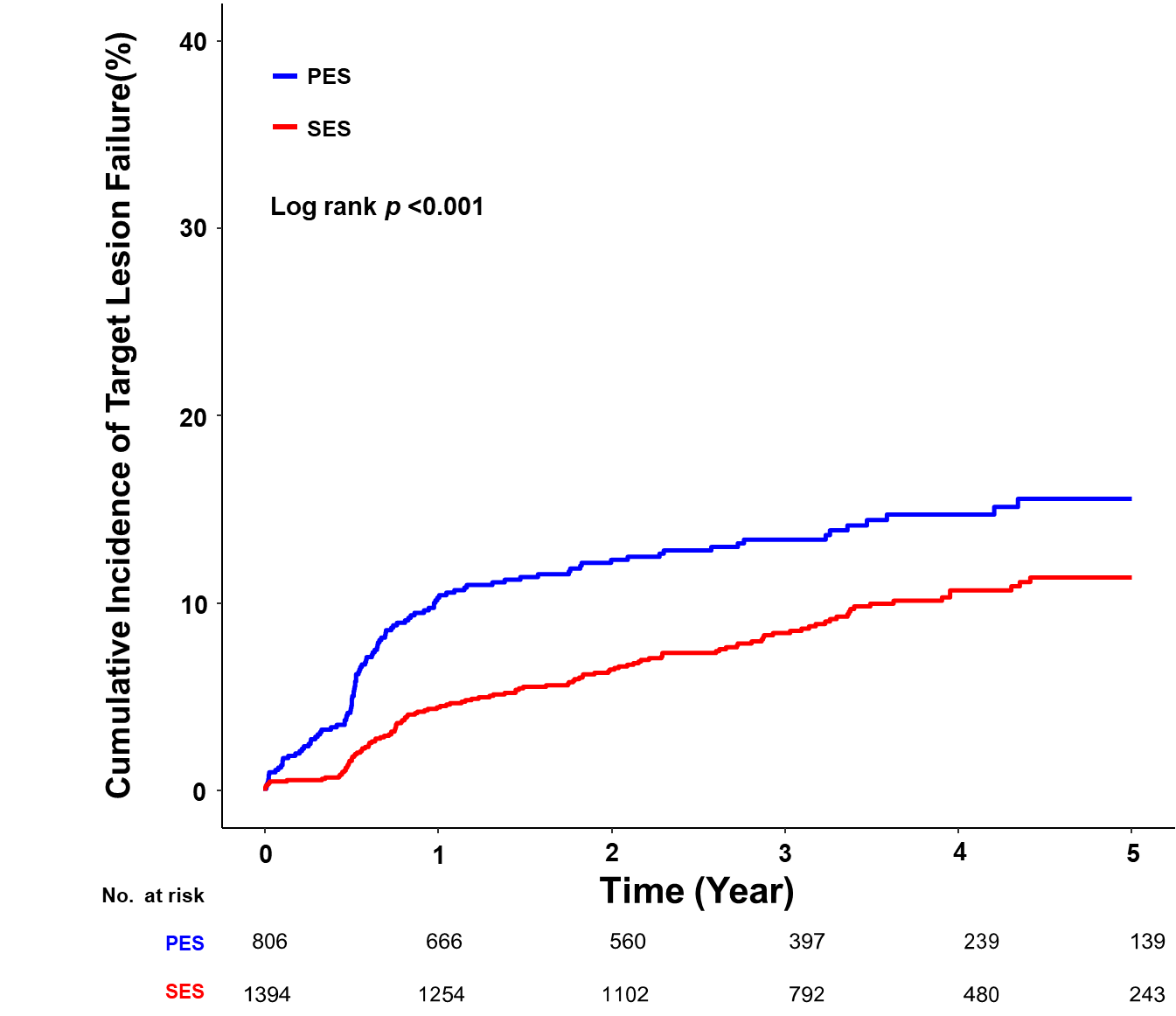
**

**Supplemental Figure 1. Comparison of 5-Year Clinical Outcomes According to Stent Type (Paclitaxel or Sirolimus-eluting First-Generation DES)**

Kaplan-Meier curves comparing the risk of target lesion failure between the use of PES and SES among patients treated with PCI for bifurcation lesions by using first-generation DES.

Abbreviations: PES, paclitaxel-eluting stent, SES, sirolimus-eluting stent.
